# Long-Term Functional Outcomes after Hand Burns: A Monocentric Controlled Cohort Study

**DOI:** 10.3390/jcm13123509

**Published:** 2024-06-15

**Authors:** Nikolaus Watzinger, Andrzej Hecker, David Petschnig, Jana Tran, Caroline Glantschnig, Maximilian Moshammer, Anna-Lisa Pignet, Anna-Maria Ellersdorfer, Lars-Peter Kamolz

**Affiliations:** 1 Division of Plastic, Aesthetic and Reconstructive Surgery, Department of Surgery, Medical University of Graz, 8036 Graz, Austrialars.kamolz@medunigraz.at (L.-P.K.); 2 COREMED—Centre for Regenerative Medicine and Precision Medicine, Joanneum Research Forschungsgesellschaft mbH, Neue Stiftingtalstraße 2, 8010 Graz, Austria

**Keywords:** burns, hand burns, functionality, PROM, long-term outcome

## Abstract

**Background**: Hand burns are involved in 80–90% of severe burn injuries. Even though hands correspond to a small total burn surface area (TBSA) of less than 5%, the loss of their functionality has a significant impact on the patient’s life. Studies that provide long-term results regarding hand functionality after hand burns are scarce. Therefore, this study aimed to investigate functional long-term results in a patient-centric approach regarding burn depth, unilateral and bilateral hand involvement, and (non-)isolated hand burns as potential influencing factors in patients with hand burns. **Methods**: We conducted a controlled cohort study of patients with burned hands treated at our department between 2005 and 2022. Healthy age-, sex-, and handedness-matched participants were used as controls. Data on the demographics, burn-related injuries, and treatments were collected. For a patient-centric approach, we used the Disabilities of the Arm, Shoulder, and Hand Questionnaire (Quick-DASH) and the Michigan Hand Questionnaire (MHQ) as patient-reported outcome measures for functional long-term evaluation, and the Patient and Observer Scar Assessment Scale (patient scale) for assessing long-term scar quality. **Results**: We enrolled 61 patients with 88 affected hands and 63 matched control participants. Up to 77.1% of the participants were male, with a mean age of 50.7 (±15.5) years and a follow-up of 8.1 (±4.7) years. The mean TBSA was 13.9% (±15.8), with 72.4% of the hands presenting with deep partial-thickness and full-thickness burns and most of the patients had only one hand affected (55.7%). The hand burn patients perceived significantly worse long-term functional scores in every domain of the MHQ as well as in the “overall function” and “work” of the Quick-DASH. Superficial hand burns negatively affected the two-handed activities of daily living (*p* = 0.013) and aesthetic appearance (*p* = 0.005) when both hands were involved. Isolated hand burns were associated with more difficulties in work performance (*p* = 0.03), whereas patients with bilateral hand involvement perceived more pain (*p* = 0.025). **Conclusions**: The patients with hand burns can achieve satisfactory long-term functional outcomes over time, but they do not reach the same long-term hand functionality as the healthy matched control group. Our study revealed that factors such as burn depth, unilateral or bilateral hand involvement, and (non-)isolated hand burns indeed have an impact on certain aspects of perceived long-term hand functionality.

## 1. Introduction

Burns represent a significant disruption in the lives of affected patients. They not only cause physical impairments but can also impact mental health and well-being [1]. With advances in the treatment of burn victims, the mortality rate associated with burns is decreasing [2]. The systematic recording and documentation of these impairments in burn survivors is essential in order to identify the possible effects on recovery [3,4]. The American Burn Association (ABA) acknowledges hand burns as serious injuries even though they do not directly impact mortality [5,6]. Therefore, special attention should be paid to hand burns, as hands are affected in 80–90% of severe burns [7,8]. Hands account for less than 5% of the total body surface area (TBSA), but the loss of their functionality has a significant impact on the body’s total functional impairment [7,9]. Everyday activities such as bathing or putting on shoes become challenging tasks [10,11]. Also, social interactions may be impaired as hands play a major role in social interaction [12].

The hand is characterized by a multitude of anatomical structures that are closely adjacent [13]. Important functional structures such as nerves, muscles, ligaments, tendons, and joints lie relatively superficially and are especially endangered [14]. Consequently, hand burns are particularly predisposed to diminished functionality [15,16]. However, known factors that mitigate the negative effects and improve rehabilitation include an early initiation of intensive exercise and physiotherapy [17,18]. Several studies have demonstrated that individuals with hand burns experience the most improvement three months after the burn incident with the implementation of physiotherapy [19,20]. Additionally, the ability to return to work significantly impacts hand functionality in a positive manner, particularly for patients with manual jobs requiring hand dexterity [6]. Conversely, factors known to adversely affect rehabilitation include surgical procedures, burn depth, and burn-associated complications such as scar contracture, pain, and wound healing disorders [6,9,21]. Wound healing disorders remain an important issue as they can increase the risk of infections and promote the proliferation of scar tissue, thereby negatively influencing the functional outcome [8]. Scars are particularly problematic, as 70% of burn survivors face the challenge of hypertrophic scarring [22]. Scarring not only affects functionality but is also associated with pain due to the reduction in nerve density in the skin [23]. Even after 18 months, stiffness and pain can persist, further negatively impacting the quality of the scars [24]. Scarring not only affects hand functionality but also poses an aesthetic issue. Hands are needed for social interaction like communication and cannot be hidden under clothing [12,14]. As a result, scars on the hands are perceived as particularly unpleasant by burn patients, which, in turn, can affect their aesthetic and psychological well-being [25]. Hence, successful burn rehabilitation is not only essential for regaining hand functionality but also for a successful holistic reintegration into society [18,20,26].

Patient-reported outcome measures (PROM) are essential for evaluating burn patients because solely conducting a clinical assessment may overlook crucial aspects of the patients’ rehabilitation and well-being [27]. To the best of the authors’ knowledge, there are no studies that provide long-term results beyond 5 years regarding hand functionality after hand burns [28]. Factors such as burn depth, hand involvement (unilateral or bilateral), and (non-)isolated hand burns indeed have an impact on certain aspects of perceived hand functionality. Even though studies on isolated hand burns and hand involvement are very sparse, these factors have already shown that they may influence hand functionality [29,30,31]. Based on this, we hypothesized that these factors impact certain aspects of long-term hand functionality in patients who have undergone hand burns. Therefore, the aim of this study was to investigate functional long-term results through a patient-centric approach, while regarding burn depth, unilateral and bilateral hand involvement, and (non-)isolated hand burns as potential influencing factors in patients with hand burns.

## 2. Materials and Methods

This monocentric, controlled cohort study was conducted between June 2022 and June 2023. It was performed in line with the principles of the Declaration of Helsinki. The institutional Ethics Committee (34-447 ex 21/22, 5 August 2022) of the Medical University of Graz gave its approval. The methods were carried out in accordance with relevant guidelines and regulations. From all the individual participants included in this study, informed consent was obtained.

### 2.1. Study Design and Patients

We searched the electronic patient records in the institutional database using the ICD-10 (International Statistical Classification of Diseases and Related Health Problems) code T23, for burn or chemical burn of the wrist and hand, to identify patients with hand burns. All patients with hand burns treated at the Division of Plastic, Aesthetic, and Reconstructive Surgery, Department for Surgery, University Hospital Graz, between 2005 and 2022, who were willing to participate, were included in this study. At our department, superficial partial thickness burns were treated with early surgical excision in combination with a skin substitute (e.g., Suprathel^®^, PolyMedics Innovations GmbH, Denkendorf, Germany), and deep partial-thickness burns with early surgical excision in combination with a split-thickness skin graft with or without a skin substitute [32]. Immediate active mobilization of the affected hand was initiated when skin substitutes (e.g., Suprathel^®^) were used following early surgical excision for deep hand burns. When skin grafts such as split-thickness skin grafts were used, active hand mobilization was initiated three to four days postoperatively. Once stable engraftment conditions were achieved, active and passive hand mobilization was conducted by our physical therapist. Additionally, in both situations, compression therapy was applied for at least one year. It is worth mentioning that our rehabilitation approach cannot be implemented in every hand burn patient with precise timing due to varying burn severity, wound conditions, and intensive care requirements. Nonetheless, early hand rehabilitation should be pursued as soon as possible [18]. The only exclusion criteria were patients under 18 years of age at the time of the hand burn. The cohort of individuals with hand burns was matched with healthy subjects who had never experienced hand-related burns, trauma, or surgery. We matched our cohorts based on sex, age, and handedness. All identified patients with hand burns and healthy subjects were contacted by phone and invited for a long-term (re-)evaluation in our outpatient setting. Patients who could not come in person received study-specific questionnaires by post. In this study, patient-reported outcomes were assessed with validated questionnaires regarding hand functionality, activities of daily living (ADL), work, aesthetic appearance, pain, and scar quality. The following patient-reported outcome measurement (PROM) questionnaires were used: the Michigan Hand Questionnaire (MHQ), Quick-DASH, and a patient scale of Patient Observer and Scar Assessment Scale (POSAS).

#### 2.1.1. Data Collections

Based on the clinical data provided in the internal hospital information system, subsequent demographic records were examined such as age of injury, age of questioning, sex, body mass index, smoking status, and handedness in our hand burn patients. Furthermore, hand burn- and injury-related data were examined (affected hand, % of total burned surface area (TBSA), burn depth, affected anatomical areas, burn reason, isolated hand burn, length of stay (LOS) in hospital and intensive care unit (ICU), inhalation trauma, and burn-related surgery). Healthy control subjects were asked about their sex, age, handedness, and whether they had experienced hand-related burns, trauma, or surgery.

#### 2.1.2. Patient Reported Outcome Measures

##### Quick-DASH

The Quick-DASH questionnaire is used for self-assessment of the functionality of the upper extremities and is validated for musculoskeletal diseases or burns [19]. It consists of 11 general questions and two subtests with five questions, each on work- and sport-related difficulties. Each answer offers up to five points (1 = no restriction, 5 = not possible). A high number of points indicates a severe restriction of the hand [19].

##### Michigan Hand Questionnaire

The MHQ is a validated questionnaire that is used for hand injuries and hand burns [11]. It evaluates the functionality of the individual hand (right/left hand) as well as both hands. The questionnaire consists of six domains, which include general hand function, specific hand function, pain, work, aesthetic appearance, and satisfaction. The higher the number of points achieved, the better the hand function (0–100), except for the pain sub-category. Here, lower scores indicate less pain. Each questionnaire evaluates both hands separately, except for the domains of pain and work [33].

##### Patient Observer and Scar Assessment Scale

The scar quality was measured with the Patient Observer and Scar Assessment Scale (POSAS). POSAS evaluates scar quality through visual and sensory perception, and palpation [34]. In this study, we exclusively analyzed the patient’s subjective view of the hand without considering the observer’s view. The patient evaluates the pain, itching, color, stiffness, thickness, irregularity, and overall impression. They can then award points ranging from 1 (e.g., no pain) to 10 (e.g., yes, had severe pain). The higher the number of points, the worse the patient-related scar quality is [34].

#### 2.1.3. Statistical Analysis

All statistical analyses were performed using IBM^®^ SPSS^®^ (Statistics ver. 28, Armonk, North Castle, NY, USA). The statistical evaluation included means or medians, standard deviations (SD) or ranges of continuous or ordered variables, and relative frequencies of categorical factors. Continuous data were examined using two-tailed *t*-tests while the chi-squared test was applied for categorical data analysis. One-way ANOVA was used to compare continuous variables, and a post hoc test with Bonferroni adjustment was subsequently used when significant main effects were present. Superficial burns were grouped with superficial (first-degree burns = 1°) and superficial partial-thickness burns (superficial second-degree burns = 2a°), and deep burns with deep partial-thickness (deep second-degree burns = 2b°) and full-thickness burns (third-degree burns = 3°). For the MHQ-based analysis of individual hand functionality, only the affected hands were considered. A two-tailed *t*-test or chi-squared test was used for group comparisons between patients with hand burns and healthy control subjects. One-way ANOVA with Bonferroni adjustment was conducted for group comparisons regarding burn depth (control vs. superficial burns vs. deep burns), hand involvement (control vs. unilateral vs. bilateral), and (non-)isolated hand burns (control vs. non-isolated vs. isolated). All statistical tests were two-tailed, and differences were considered statistically significant when *p* < 0.05.

## 3. Results

### 3.1. Patient Characteristics

Out of a total of 299 potential patients with hand burns who were identified as eligible for study inclusion, only 61 (20.4%) patients agreed to participate in this study. Among the included 61 patients with hand burns, there were 14 women (22.9%) and 47 men (77.1%). The mean age at the time of the hand burn-related accident was 43.0 years (SD ± 16.8), whereas the mean age at the time of examination was 50.7 years (SD ± 15.5). A demographic overview of our patient cohort with burned hands is given in Table 1. We included 63 healthy control subjects, consisting of 30.2% women (*n* = 19) and 69.8% men (*n* = 44). Here, the mean age was 49.9 (SD ± 14.9) years at the time of examination. There were no significant differences between our hand burn group and the healthy matched control group (CG) regarding age, gender and handedness (Table 1).

### 3.2. Hand-Related Burn Characteristics

In total, 88 burned hands from 61 patients were enrolled in this study, with 80.3% of the patients having their dominant hand affected. Flames (43.3%) were the most common reason for hand burn injuries, followed by hot oil (15%) and explosions (13.3%). Up to 83.6% of the patients had a non-isolated hand burn, with the head/face (47.5%) and forearm (45.9%) being the most affected areas. In more than half of all the cases (55.7%), a unilateral hand burn was reported. A total of 78.3% of these hand burns presented as deep partial-thickness or full-thickness burns. Table 2 provides a detailed overview of the characteristics of hand burns.

### 3.3. Results of Patient Related Outcome Measures

#### 3.3.1. Functionality (Quick-DASH)

**Figure 1:**
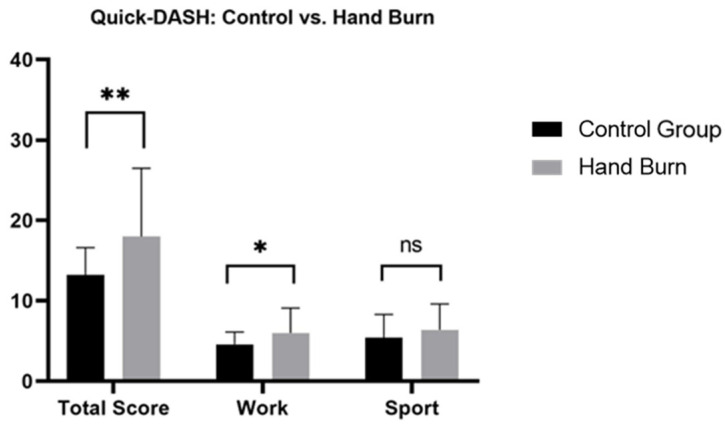
Long-term Quick-DASH scores comparison between patients with hand burns and healthy matched control group. Quick-DASH: Quick disability of Arm, Shoulder and Hand; ns: not significant. A two-tailed *t*-test was used. * *p* < 0.05; ** *p* < 0.001.

**Figure 2:**
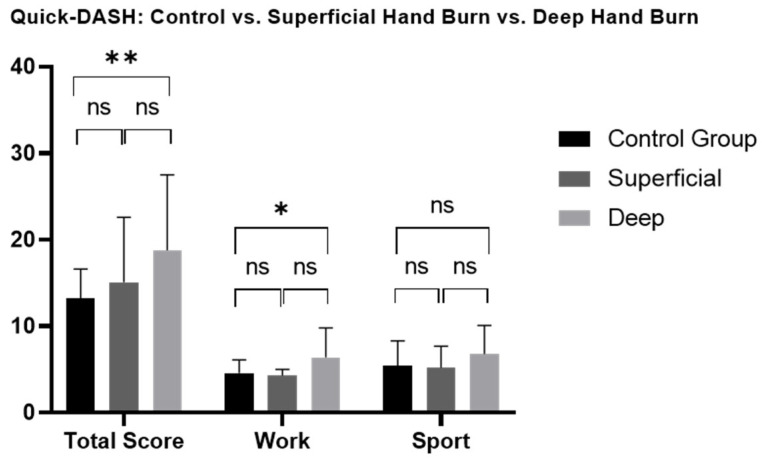
Long-term Quick-DASH scores comparison between patients with superficial and deep hand burns and healthy matched control group. Quick-DASH: Quick disability of Arm, Shoulder and Hand; ns: not significant. One-way ANOVA with Bonferroni adjustment was used. * *p* < 0.05; ** *p* < 0.001.

**Figure 3:**
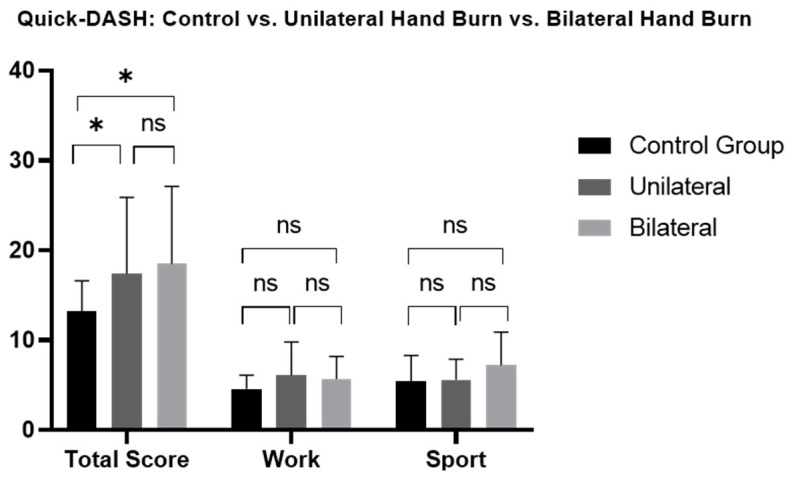
Long-term Quick-DASH scores comparison between patients with unilateral and bilateral hand burns and healthy matched control group. Quick-DASH: Quick disability of Arm, Shoulder and Hand; ns: not significant. One-way ANOVA with Bonferroni adjustment was used. * *p* < 0.05.

**Figure 4:**
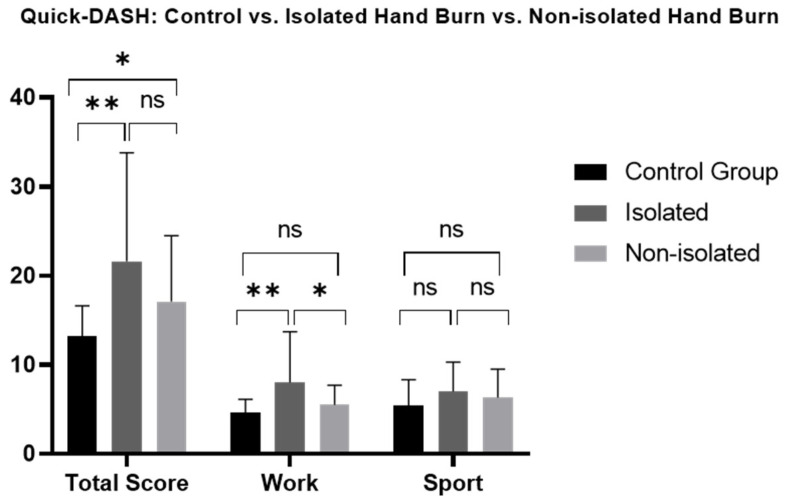
Long-term Quick-DASH scores comparison between patients with isolated and non-isolated hand burns and healthy matched control group. Quick-DASH: Quick disability of Arm, Shoulder and Hand; ns: not significant. One-way ANOVA with Bonferroni adjustment was used. * *p* < 0.05; ** *p* < 0.001.

Patients with hand burns showed significantly impaired long-term hand functionality and work-related abilities (Figure 1, Table S1). Deep hand burns, unilaterally and bilaterally affected hands, and isolated or non-isolated hand burns, showed a significant long-term impairment in functionality compared to the CG, where no significance was found in superficial hand burns. No significant differences were found in total functionality scores or sport-related functional scores based on burn depth (superficial/deep), hand involvement (unilateral/bilateral), and hand isolation (isolated/non-isolated hand burn) (Figure 2, Figure 3 and Figure 4, Tables S2–S4).

#### 3.3.2. Functionality and Activities of Daily Living (MHQ)

**Figure 5:**
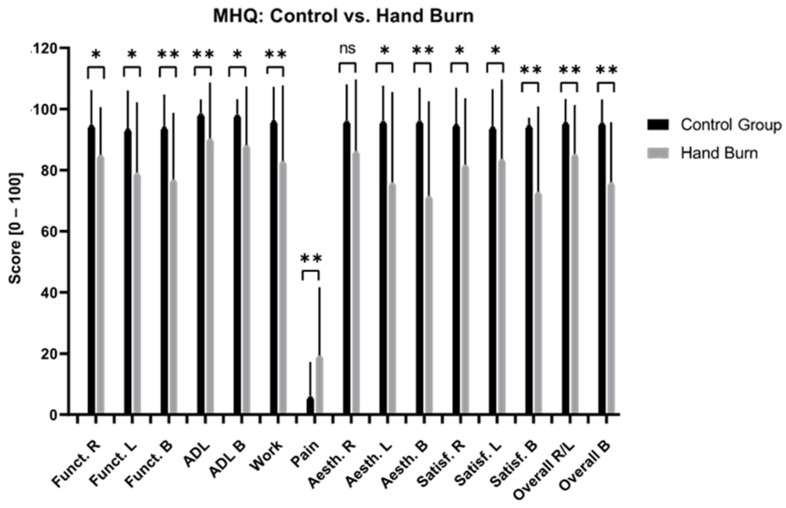
Long-term MHQ scores comparison between patients with hand burns and healthy matched control group. MHQ: Michigan Hand Questionnaire; R: right hand; L: left hand; B: both hands; Funct.: function; ADL: activities of daily living; Aesth.: aesthetic; Satisf.: satisfaction; ns: not significant. A two-tailed *t*-test was used. * *p* < 0.05; ** *p* < 0.001.

**Figure 6:**
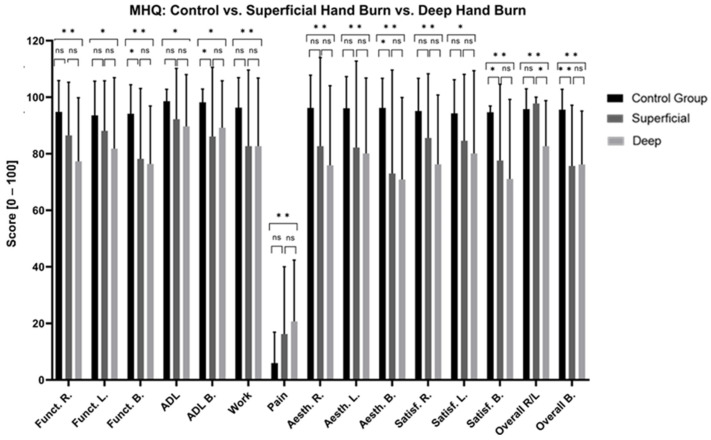
Long-term MHQ scores comparison between patients with superficial and deep hand burns and healthy matched control group. MHQ: Michigan Hand Questionnaire; R: right hand; L: left hand; B: both hands; Funct.: function; ADL: activities of daily living; Aesth.: aesthetic; Satisf.: satisfaction; ns: not significant. One-way ANOVA with Bonferroni adjustment was used. * *p* < 0.05; ** *p* < 0.001.

**Figure 7:**
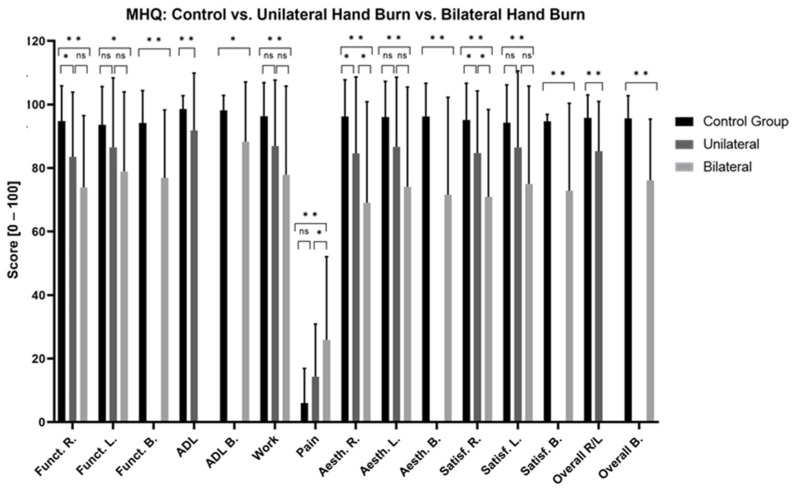
Long-term MHQ scores comparison between patients with unilateral and bilateral hand burns and healthy matched control group. MHQ: Michigan Hand Questionnaire; R: right hand; L: left hand; B: both hands; Funct.: function; ADL: activities of daily living; Aesth.: aesthetic; Satisf.: satisfaction; ns: not significant. One-way ANOVA with Bonferroni adjustment and a two-tailed *t*-test were used. * *p* < 0.05; ** *p* < 0.001.

**Figure 8:**
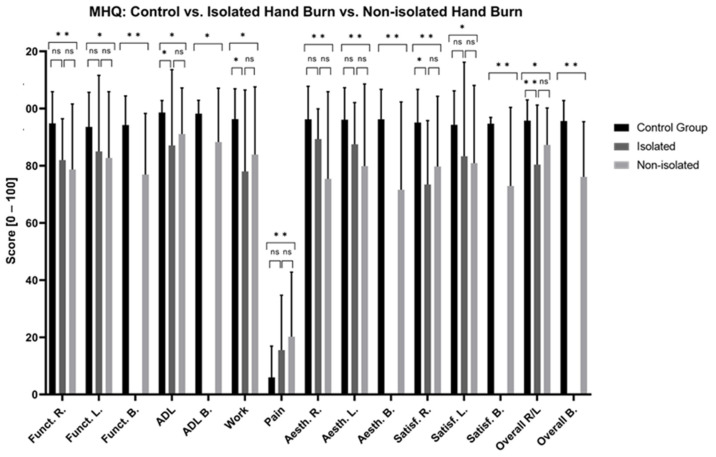
Long-term MHQ scores comparison between patients with isolated and non-isolated hand burns and healthy matched control group. MHQ: Michigan Hand Questionnaire; R: right hand; L: left hand; B: both hands; Funct.: function; ADL: activities of daily living; Aesth.: aesthetic; Satisf.: satisfaction; ns: not significant. One-way ANOVA with Bonferroni adjustment and a two-tailed *t*-test were used. * *p* < 0.05; ** *p* < 0.001.

Patients with hands affected by burns presented significantly poorer long-term functionality and greater difficulties with ADL compared to the CG (Figure 5, Table S1). Superficial and deep burns, unilateral burns of the right hand, bilaterally affected hands, and non-isolated hand burns significantly impaired long-term hand functionality (Figure 6, Figure 7 and Figure 8, Tables S2–S4). No significant differences were found in the MHQ functionality scores based on burn depths (superficial/deep burn), hand involvement (unilateral/bilateral) and hand isolation (isolated/non-isolated hand burn). Regardless of burn depth, hand involvement, and (non-)isolation, the results show significantly impaired hand functionality regarding activities of daily living compared to the CG (Figure 5, Figure 6, Figure 7 and Figure 8, Tables S2–S4).

#### 3.3.3. Aesthetic and Satisfaction (MHQ)

Patients with hand burns perceive their hand as aesthetically and satisfactorily worse compared to the CG after the long-term follow-up (Figure 5, Table S1). Deep hand burns, bilateral hand involvement, and non-isolated hand burns presented significantly worse long-term aesthetic and satisfaction scores compared to the CG. Superficial burns showed significantly worse scores in hand aesthetics (*p* = 0.005) and satisfaction (*p* = 0.03) when both hands were affected. Patients with unilateral burns on their right hand perceived their hand aesthetic as significantly more appealing compared to bilateral hand involvement (*p* = 0.015). Isolated hand burns negatively affect long-term satisfaction scores of the right hand (*p* = 0.003), but not the left hand, nor do they affect hand aesthetic scores (Figure 5, Figure 6, Figure 7 and Figure 8, Tables S2–S4).

#### 3.3.4. Pain (MHQ)

Patients with burned hands reported significantly more pain compared to the CG (Figure 5, Table S1). Patients with deep hand burns (*p* < 0.001), bilaterally affected hands (*p* < 0.001), and non-isolated hand burns (*p* < 0.001) showed significantly worse pain scores compared to the CG. Patients with bilaterally affected hands reported significantly worse pain scores compared to patients with unilaterally affected hands (*p* = 0.025) (Figure 5, Figure 6, Figure 7 and Figure 8, Tables S2–S4).

#### 3.3.5. Work (Quick-DASH, MHQ)

At the time of examination, 7 patients (11.5%) were already retired, 40 (65.6%) were still working, and 14 (22.9%) did not provide any information regarding their employment status. In total, 50 (80.2%) patients stayed at their old profession, whereas 8 (13.8%) patients changed their job after the hand burn-related injury. Among these eight patients, one had retired (12.5%) and seven (87.5%) were still working in different jobs at the time of the examination. In general, patients with hand burns have significantly more long-term work-related issues compared to the CG (Figure 1 and Figure 5, Table S1). Patients with deep hand burns presented significant work-related functional impairments in the Quick-DASH (*p* = 0.003) (Figure 2, Table S2). Those with isolated hand burns encountered more long-term work-related functional difficulties than non-isolated hand burns (*p* = 0.030) and the CG (*p* < 0.001) (Figure 4, Table S4). Deep burns, bilateral hand involvement, and isolated and non-isolated hand burns showed significantly worse work-related performance compared to the CG in the MHQ (Figure 5, Figure 6, Figure 7 and Figure 8, Tables S2–S4).

#### 3.3.6. Scar Quality (POSAS, Patient Perspective)

No significant differences were found in the total patient-reported POSAS scores regarding burn depth (*p* = 0.172), hand involvement (*p* = 0.417), and (non-)isolated hand burns (*p* = 0.507). Furthermore, no significant differences in the sub-categories of the POSAS could be found (Table S5).

## 4. Discussion

Hand burns frequently occur either as components of extensive burns or as isolated injuries and can cause significant functional impairment [26]. However, the functional recovery of burn survivors does not end upon the discharge of the hospital, but rather, it fluctuates over time [35]. Based on that, past studies have revealed that occupational therapy and early stages of rehabilitation therapy are key factors for achieving the best functional results [18,20]. However, previous studies have evaluated hand burn-based outcomes with a long-term follow-up for only up to five years [6,18,28,31]. To the best of the authors’ knowledge, this is the first study that provides long-term results above 5 years regarding hand functionality after hand burns [28]. In contrast, our study evaluated patients’ perceived hand functionality after a mean long-term follow-up of 8 years. We only used a patient-centric approach with PROMs in this study to evaluate the patients’ self-perspective regarding the long-term functional results. These data can give crucial insights into patients’ rehabilitation and well-being [27]. Additionally, our hand burn cohort was compared to a healthy sex-, age-, and handedness-matched control cohort. Compared to our CG, the hand burn cohort reported significantly worse scores in every domain of the MHQ (function, pain, ADL, aesthetic, satisfaction, overall) as well as in the overall function and work domains of the Quick-DASH. No sports-related perceived functional impairments could be found. Our study revealed that factors such as burn depth, unilateral or bilateral hand involvement, and (non-)isolated hand burns indeed have an impact on certain aspects of perceived hand functionality. Even though studies on isolated hand burns and hand involvement are very sparse, these factors have already shown that they may influence hand functionality [29,30,31]. In the following sections, our long-term results regarding hand functionality, ADL, work, scar quality, aesthetic appearance, and pain will be discussed.

### 4.1. Hand Functionality

Deep burns not only entail a prolonged recovery period but also result in the destruction of underlying anatomical structures, thereby compromising functionality [16,31]. This often leads to longstanding functionality impairments of the affected hands up to 12 months after the accident [31]. However, after 5 years, satisfying to normal long-term hand functionality after deep partial- and full-thickness hand burns has been reported by Cartotto et al. [28]. Compared to the healthy subjects in our study, deep (*p* < 0.001) and superficial (*p* = 0.013) hand burns impaired two-handed long-term functionality, whereas one-handed functionality was only negatively affected by deep burns (Table S2). Even though there is a difference from a statistical point of view, one-hand and two-handed functionality in the MHQ were considered good in our hand burn cohort for both superficial (right hand: 86.5, left hand: 88.1, both hands: 78.2) and deep burns (right hand: 77.3, left hand: 81.8, both hands: 76.4). Interestingly, no functionality differences could be found between the superficial and deep hand burns for both the one- and two-handed functionalities. The comparable and generally low TBSA of superficial (11.4% ± 8.7) and deep hand burns (14.6% ± 17.3) may play a role here (*p* = 0.555). Taking into account that severe burns are typically defined by a TBSA as exceeding 20% [36], the TBSA within our study cohort was not only low (13.9%), but notably lower when compared to other studies focused on hand burns (TBSA: 22–58%) [28,37]. Furthermore, none of our patients’ extensor mechanisms, joint capsules, or bones were affected by deep burns. Even though this type of injury is very rare, accounting for only 5% of all hand burns, it is often accompanied by significant functional impairment [30]. It is worth mentioning that through the evolution of burn care in the last decade, significant advances have contributed to current standards of care in burn management and ultimately improved patient outcomes [38]. Especially in the last decade, a rapid improvement in skin substitute development has been reported, whereby skin substitutes have shown comparable long-term results in scar quality compared to autologous skin donor sites [32,39]. These aspects could contribute to the comparable perceived functional long-term results regarding burn depth. However, Sheridan et al. [30] reported contrary results regarding functionality in 1047 hand burns. This study showed that 81% of deep partial- and full-thickness hand burns regained normal hand functionality, whereas almost all superficial burns (97%) regained complete hand function [30]. In this study as well, the patients exhibited a higher average TBSA compared to our hand burn cohort (23.3% vs. 13.9%), further emphasizing the significant impact of deep hand burns in combination with higher TBSA on hand functionality.

Based on the nature of non-isolated hand burns, patients experienced more severe injuries regarding burn depth and area compared to isolated hand burns. Thus, non-isolated hand burns present with more severe burn extension, which ultimately negatively affects functional outcome [40,41]. Similar results could be found in our hand burn cohort. Here, non-isolated hand burns showed significantly impaired one-handed and two-handed functionality compared to the CG, whereby patients with isolated hand burns (right hand: *p* = 0.092, left hand: *p* = 0.599) presented comparable results. Additionally, non-isolated hand burns are associated with higher TBSA [40], thus more body parts are potentially affected. This does not only lead to a longer inpatient stay [40] but can also lead to more concerning emergency procedures or intensive care treatment in these patients [8,42]. As those are the primary preferences after severe burns, consequently, the affected hands may be neglected because their impairment is perceived as less life-threatening [8]. However, 27.9% of our burn patients remained in the ICU with a mean length of stay of 11.8 days. Hand functionality of our ICU patients and non-ICU patients were comparable (Quick-DASH: *p* = 0.762, MHQ: *p* = 0.293), thus an ICU stay did not affect the perceived long-term functional results in our hand burn cohort. However, early surgery is associated with better hand functionality compared to delayed surgery and should be considered in a setting of intensive care [16]. Moreover, the functionality between isolated and non-isolated hand burns were comparable (*p* = 1.000) in our hand burn group. These findings align with those of Aghajanzade et al. [43], showing similar results between isolated and non- isolated hand burns. A contributing factor for our good results in non-isolated hand burns could be the improved alignment with the situation, as described by Renneberg et al. [44], among patients with severe burns. Here, patients with non-isolated hand burns showed a well-established coping mechanism to manage the trauma and disability. Social support and self-efficacy were adaptive strategies to overcome these obstacles [44]. Nevertheless, these results must be interpreted with caution, as our study only included ten isolated hand burns.

However, non-isolated hand burns not only present larger TBSA but also more frequently exhibit bilateral hand involvement, with rates of up to 35% compared to 9% of isolated hand burns [40]. Bilateral hand involvement not only correlates with a higher number of surgeries [45] but also leads to poorer hand functionality [29]. Additional surgeries frequently cause further anatomical hand alterations, leading to increased functional impairment [41]. In contrast with these findings, hand functionality was comparable between our patients with unilateral and bilateral hand involvement (Table S3). Although the proportion of bilateral hand involvement was similar to that which was reported by Li et al. [29], relatively more patients underwent surgery (90.5%) compared to our hand burn cohort (70.5%). These aspects likely led to comparable functional results between the unilaterally and bilaterally involved patients in our hand burn cohort.

### 4.2. Activities of Daily Living

Alongside impaired hand function, activities of daily living (ADL) such as the tying of shoelaces can be challenging for patients after hand burns [18]. Even one year after the injury, hand burn patients showed difficulties concerning ADL [18]. ADL can be influenced by burn-related amputations and deep burns [37]. Concordant with hand functionality, deep burns also negatively affected the perceived one-handed and two-handed ADL in our hand burn cohort. Interestingly, superficial hand burns only negatively affected two-handed ADL (*p* = 0.013), but not one-handed ADL (*p* = 0.330). Both isolated and non-isolated hand burns impaired ADL in our hand burn cohort, but they were comparable to each other, mirroring the results concerning hand functionality. The patients’ perceptions of hand function in our study cohort corresponded with their abilities to actually perform most of the ADL. Just as with long-term hand functionality, the ADL scores were good in our hand burn cohort (one-handed ADL: 90.4 ± 18.0; two-handed ADL: 88.3 ± 18.8). In our study, most of the patients (94%) with hand burns returned to work after the hand burn-related injury. Taking into account that recovery of hand functionality aligns with the ability to return to work [41,46], this could be a reasonable explanation of our good functional long-term results.

### 4.3. Working Capacity

Although most of our patients returned to work after their hand burns, these patients presented significant perceived long-term work-related performance issues (Quick-DASH: *p* = 0.021, MHQ: *p* < 0.001). Additionally, patients with deep burns and bilateral hand affection seem to perceive worse long-term work performance (respectively, *p* < 0.001). Deeper burns and bilateral hand involvement often present with more severe burns, thus they are more likely to be associated with increased scarring. This often leads to a reduction in functionality, which can lead to impaired work performance [47]. Although no statistical differences were observed regarding the patients’ perceived scar quality, descriptively worse scores were found in deep and bilateral hand burns compared to superficial burns or unilateral hand involvement (Table S5). Patients with isolated hand burns also perceived work-related performance impairments (Quick-Dash: *p* < 0.001, MHQ: *p* = 0.015). Interestingly, isolated hand burns even showed significantly worse work-related scores compared to non-isolated hand burns (*p* = 0.030). Patients with isolated or unilateral hand burns may tend to rely more on the healthy hand, thereby neglecting the injured hand, whereas those with bilaterally affected hands are compelled to utilize the burned hand. Derived from this explanation, a reduced use of the affected hand could potentially be responsible for the poorer work performance [48]. Regarding the process of recovery, having a job before the burn injury might positively affect the outcome and post-burn employment for patients [4]. It has also been stated that patients who were employed after their burn injuries had higher scores in physical and social functioning, bodily pain, and mental health, compared to the unemployed patients [49]. However, in our study, these possible correlations were not further investigated.

### 4.4. Scar Quality, Aesthetic Appearance, and Pain

Our study cohort presented satisfactory perceived scar quality with no significant long-term differences regarding unilateral or bilateral hand involvement, (non-)isolated hand burns, and burn depth. However, Spronk et al. [25] reported that patients perceived the scar appearance in burned areas as distinctly different from that of unaffected skin more than 5 years after their burn injuries. Interestingly, there was no significant difference in all aspects of scar quality (pain, itching, color, stiffness, thickness, irregularity, patients’ overall opinion) in our hand burn cohort regarding burn depth, unilateral or bilateral hand involvement, and (non-)isolated hand burns (Table S5). Scar quality differences were more pronounced in patients with severe burns (>20%) compared to those with mild or moderate burns (<10% TBSA) [25]. Taking that into account, our results could be explained by the fact that a lesser burn extent was reported in our hand burn cohort (TBSA 13.9%), and here, the patient-related scar assessment of only the affected hands rather than of other affected body parts was conducted. However, scar quality following hand burns can significantly impact not only hand functionality but also the aesthetic perception of the burned area [50]. In our study, patients with a unilaterally affected right hand perceived their hand aesthetic as significantly better compared to patients with bilaterally affected hands (*p* = 0.015), whereby isolated hand burns do not seem to have an impact here. A possible explanation could be that patients with unilateral hand burns have a healthy hand to compare against as they observe the healing process. But for those with burns on both hands, it is harder to maintain the same point of reference. This concept of a superimposed comparison is used in mirror therapy [51]. Interestingly, even superficial hand burns showed a noticeable impact on patients’ aesthetic concerns, when both hands were affected. Nonetheless, the quality of scarring after burns not only affects the aesthetic appearance but can also be associated with pain, and particularly with poorer scar quality [25]. Our hand burn patients perceived significantly more long-term pain compared to the healthy group, especially patients with deep hand burns (*p* < 0.001), bilaterally affected hands (*p* < 0.001), or non-isolated hand burns (*p* < 0.001). These results are not surprising, as severe burns are associated not only with increased scarring but also with more pain [52]. Pain can lead to an impairment of the hands and thus a reduction in their usage. Since hands are necessary for self-care, communication, occupational skills, and even emotional touch, pain should be a significant consideration in the multifactorial treatment of hand burns [53]. When dealing with hand burns, these results should help provide the respective physicians with evidence-based information about realistic convalescence expectations. Our findings indicate that patients with deeper or isolated hand burns have worse work-related outcomes, necessitating proper clarification as it might impact patients’ future work decisions. Furthermore, in patients with bilateral hand burns, special attention should be given to a comprehensive pain management strategy that addresses pain from multiple perspectives, as these patients experience more pain than those with unilateral hand burns. Finally, patients must be informed about the realistic expectations regarding possible scar formation, as we have observed that even superficial hand burns can lead to significant aesthetic impairment. Although our results offer valuable insights into significant aspects of patients’ perceptions regarding certain areas of functionality after hand burns, this study has some limitations and the necessity of controlled prospective long-term studies persists. As this is the first study to examine long-term functional outcomes concerning burn depth, unilateral/bilateral hand involvement, and isolated /non-isolated hand burns, these data can be used as power analyses for future short-term or long-term hand burn studies. Future research should focus on these subgroups in a prospective, controlled manner to obtain more information regarding reasons for functional differences in certain aspects of hand functionality and potential confounding factors in functional hand recovery, and finally identify potential areas for personalized treatment.

## 5. Limitations

This study has several limitations, including the retrospective retrieval of clinical data (e.g., variations in documentation styles, missing data), wide distribution of follow-up time, and the relatively small cohort. Of a total of 299 potential patients with hand burns, only 61 (20.4%) agreed to participate in this study. This leaves a bias, as patients who were not satisfied with their burn-related outcome might have been reluctant to participate in a hand burn study. Therefore, only limited inferences can be drawn regarding the subjective perception of hand function in our hand burn cohort. For an adequate sub-analysis of our hand burn cohort, the small cohort size further limits its statistical power. In particular, the results of isolated hand burns, which involved only 10 patients, should be interpreted with the relevant level of caution. It is important to mention that our study was a controlled cohort study, therefore it is not possible to draw conclusions regarding cause and effect. Additionally, we only used a patient-centric approach with PROMs in this study to evaluate the patients’ perspective regarding functional long-term results. Without objective data, our findings must be interpreted within this framework of limitations.

## 6. Conclusions

This study demonstrates that patients with hand burns can attain satisfactory perceived functional outcomes over an extended period. However, our hand burn group was unable to achieve the same level of long-term hand functionality as a healthy comparison cohort. Our study revealed that factors such as burn depth, unilateral or bilateral hand involvement, and (non-)isolated hand burns indeed have an impact on certain aspects of perceived hand functionality. By comparing hand isolation, the extent of hand involvement, and burn depth, we were able to demonstrate that deeper burns and isolated hand burns resulted in worse outcomes regarding work-related issues, while patients with bilateral hand burns experienced more pain than those with unilateral hand burns. Patients with superficial hand burns exhibited noticeable long-term aesthetic concerns when both hands were affected, as well as long-term difficulties with two-handed ADL. These findings should help physicians properly advise patients with hand burns so that they have realistic expectations about their recovery. Additionally, these insights could contribute to providing more targeted psychosocial support for patients facing long-term aesthetic concerns or difficulties in daily activities due to their hand burns.

## Figures and Tables

**Table 1:** Demographic characteristics of patients with hand burns.

Parameter	Hand Burn Group	Healthy Control Group	*p*-Value
Gender, *n* (%)			
Male:	14 (22.9)	19 (30.2)	0.364 ^a^
Female:	47 (77.1)	44 (69.8)	
Age at examination, (years), mean (SD)	50.7 (±15.5)	49.9 (±14.9)	0.776 ^b^
Age at accident, (years), mean (SD)	43.0 (±16.8)	NA	
Dominant hand, *n* (%)			0.528 ^a^
Right hand:	53 (86.9)	57 (90.5)	
Left hand:	8 (13.1)	6 (9.5)	
Follow–up (years), mean (SD)	8.1 (±4.7)	NA	
BMI * (kg/m^2^), mean (SD)	26.4 (±3.6)		
Smoker *, *n* (%)			
Yes	15 (25.4)		
No	44 (74.6)		

SD: standard deviation; BMI: body mass index. NA: not applicable. * no BMI and smoker data were obtained from healthy control group. ^a^ chi-squared test, ^b^ two-tailed *t*-test.

**Table 2:** Characteristics of hand burns.

Hand Burn Characteristics	Values
Burn reasons, *n* (%)	
Flames	26 (43.3)
Hot oil	9 (15)
Explosion	8 (13.3)
Contact burn	5 (8.3)
Electrical	5 (8.3)
Hot water	2 (3.4)
Chemical	2 (3.4)
Other	3 (5.0)
TBSA (total body), mean	
% (SD)	13.9 (±15.8)
% (range)	13.9 (0.2–70)
Affected areas, *n* (%)	
Head/face	29 (47.5)
Neck	9 (14.8)
Thorax	9 (14.8)
Back	6 (9.8)
Upper arm	14 (22.9)
Forearm	28 (45.9)
Abdomen	3 (4.9)
Buttocks	3 (4.9)
Thigh	13 (21.3)
Shank	15 (24.6)
Feet	10 (16.4)
Affected hands, *n* (%)	
Total	88 (100)
Unilateral (right hand)	21 (34.4)
Unilateral (left hand)	13 (21.3)
Bilateral	27 (44.3)
Handedness of burned hand, *n* (%)	
Dominant hand	49 (80.3)
Non-dominant hand	12 (19.7)
Burn depth, *n* (%)	
Superficial burn (1°–2a°)	24 (27.6)
Superficial (1°)	1 (1.2)
Superficial partial-thickness (2a°)	23 (26.4)
Deep burn (2b°–3°)	63 (72.4)
Deep partial-thickness (2b°)	34 (39.1)
Full-thickness (3°)	29 (33.3)
Isolated hand burn, *n* (%)	
Yes	10 (16.4)
No	51 (83.6)
Surgery, *n* (%)	
Yes (patients)	43 (70.5)
Yes (hands)	60 (68.2)
No (patients)	18 (29.5)
No (hands)	28 (31.8)
In-patient length of stay (days), mean (SD)	14.6 (±12.9)
ICU, *n* (%)	
Yes	17 (27.9)
No	44 (72.1)
ICU length of stay (days), mean (SD)	11.8 (±11.2)
Inhalation trauma, *n* (%)	
Yes	7 (11.5)
No	54 (88.5)

TBSA: total body surface area; SD: standard deviation; ICU: intensive care unit.

## Data Availability

The original contributions presented in the study are included in the article/supplementary material, further inquiries can be directed to the corresponding author.

## Supplementary Materials

The following supporting information can be downloaded at: https://www.mdpi.com/article/10.3390/jcm13123509/s1, Table S1: Long-term Quick-DASH and MHQ scores comparison of healthy matched control group between patients with hand burns; Table S2: Long-term Quick-DASH and MHQ scores comparison of healthy matched control group between patients with superficial and deep hand burns; Table S3: Long-term Quick-DASH and MHQ scores comparison of healthy matched control group between patients with bilaterally and unilaterally affected hand burns; Table S4: Long-term Quick-DASH and MHQ scores comparison of healthy matched control group between patients with isolated and non-isolated hand burns; Table S5: Long-term POSAS scores (patient perspective) comparison regarding burn depth, affected hands and (non-)isolated hand burns in patients after hand burns.

## References

[1] Spronk I., Legemate C., Oen I., van Loey N., Polinder S., van Baar M. (2018). Health related quality of life in adults after burn injuries: A systematic review. PLoS ONE.

[2] Brusselaers N., Hoste E.A.J., Monstrey S., Colpaert K.E., De Waele J.J., Vandewoude K.H., Blot S.I. (2005). Outcome and changes over time in survival following severe burns from 1985 to 2004. Intensive Care Med..

[3] Smolle C., Hutter M.F., Kamolz L.P. (2022). Life after Burn, Part II: Substance Abuse, Relationship and Living Situation of Burn Survivors. Medicina.

[4] Hutter M.F., Smolle C., Kamolz L.P. (2022). Life after Burn, Part I: Health-Related Quality of Life, Employment and Life Satisfaction. Medicina.

[5] Edger-Lacoursière Z., Deziel E., Nedelec B. (2023). Rehabilitation interventions after hand burn injury in adults: A systematic review. Burns.

[6] Knight A., Wasiak J., Salway J., O’Brien L. (2017). Factors predicting health status and recovery of hand function after hand burns in the second year after hospital discharge. Burns.

[7] Bache S.E., Fitzgerald O’Connor E., Drake P.J.H., Philp B., Dziewulski P. (2018). Development and validation of the Burnt Hand Outcome Tool (BHOT): A patient-led questionnaire for adults with hand burns. Burns.

[8] Kamolz L.P., Kitzinger H.B., Karle B., Frey M. (2009). The treatment of hand burns. Burns.

[9] Schneider J.C., Holavanahalli R., Helm P., O’Neil C., Goldstein R., Kowalske K. (2008). Contractures in Burn Injury Part II: Investigating Joints of the Hand. J. Burn Care Res..

[10] Schneider J.C., Qu H.D., Lowry J., Walker J., Vitale E., Zona M. (2012). Efficacy of inpatient burn rehabilitation: A prospective pilot study examining range of motion, hand function and balance. Burns.

[11] Lin S.Y., Chang J.K., Chen P.C., Mao H.F. (2013). Hand function measures for burn patients: A literature review. Burns.

[12] Kinzl J. (2008). Die Hand—Ausdrucksorgan für Beziehung, Kreativität und Bewältigung. Handchir. Mikrochir. Plast. Chir..

[13] Teo W.Z.W., Chung K.C. (2019). Hand Infections. Clin. Plast. Surg..

[14] Germann G. (2017). Hand Reconstruction After Burn Injury. Clin. Plast. Surg..

[15] Pruksapong C., Burusapat C., Hongkarnjanakul N. (2020). Efficacy of Silicone Gel versus Silicone Gel Sheet in Hypertrophic Scar Prevention of Deep Hand Burn Patients with Skin Graft: A Prospective Randomized Controlled Trial and Systematic Review. Plast. Reconstr. Surg. Glob. Open.

[16] Omar M.T.A., Hassan A.A. (2011). Evaluation of hand function after early excision and skin grafting of burns versus delayed skin grafting: A randomized clinical trial. Burns.

[17] Paratz J.D., Stockton K., Plaza A., Muller M., Boots R.J. (2012). Intensive exercise after thermal injury improves physical, functional, and psychological outcomes. J. Trauma Acute Care Surg..

[18] Kara S. (2023). Effectiveness of early rehabilitation in hand burns. Turk. J. Trauma Emerg. Surg..

[19] Wu A., Edgar D.W., Wood F.M. (2007). The QuickDASH is an appropriate tool for measuring the quality of recovery after upper limb burn injury. Burns.

[20] Ghalayini G., O’Brien L., Bourke-Taylor H.M. (2019). Recovery in the first six months after hand and upper limb burns: A prospective cohort study. Aust. Occup. Ther. J..

[21] Gojowy D., Kauke M., Ohmann T., Homann H.H., Mannil L. (2019). Early and late-recorded predictors of health-related quality of life of burn patients on long-term follow-up. Burns.

[22] Kamolz L.P., Hecker A. (2023). Molecular Mechanisms Related to Burns, Burn Wound Healing and Scarring. Int. J. Mol. Sci..

[23] Isoardo G., Stella M., Cocito D., Risso D., Migliaretti G., Cauda F., Palmitessa A., Faccani G., Ciaramitaro P. (2012). Neuropathic pain in post-burn hypertrophic scars: A psychophysical and neurophysiological study. Muscle Nerve.

[24] Goei H., van der Vlies C.H., Tuinebreijer W.E., van Zuijlen P.P.M., Middelkoop E., van Baar M.E. (2017). Predictive validity of short term scar quality on final burn scar outcome using the Patient and Observer Scar Assessment Scale in patients with minor to moderate burn severity. Burns.

[25] Spronk I., Polinder S., Haagsma J.A., Nieuwenhuis M., Pijpe A., van der Vlies C.H., Middelkoop E., van Baar M.E. (2019). Patient-reported scar quality of adults after burn injuries: A five-year multicenter follow-up study. Wound Repair. Regen..

[26] Anzarut A., Chen M., Shankowsky H., Tredget E.E. (2005). Quality-of-Life and Outcome Predictors following Massive Burn Injury. Plast. Reconstr. Surg..

[27] Griffiths C., Armstrong-James L., White P., Rumsey N., Pleat J., Harcourt D. (2015). A systematic review of patient reported outcome measures (PROMs) used in child and adolescent burn research. Burns.

[28] Cartotto R. (2005). The Burned Hand: Optimizing Long-term Outcomes with a Standardized Approach to Acute and Subacute Care. Clin. Plast. Surg..

[29] Lin S.Y., Chen C.C., Mao H.F., Hsiao F.Y., Tu V.Y.H. (2013). The development and preliminary validation of the Taiwanese Manual Ability Measure for Burns. Burns.

[30] Sheridan R.L., Hurley J., Smith M.A., Ryan C.M., Bondoc C.C., Quinby W.C., Tompkins R., Burke J. (1995). The Acutely Burned Hand. J. Trauma: Inj. Infect. Crit. Care.

[31] Williams N., Stiller K., Greenwood J., Calvert P., Masters M., Kavanagh S. (2012). Physical and Quality of Life Outcomes of Patients with Isolated Hand Burns—A Prospective Audit. J. Burn Care Res..

[32] Selig H.F., Keck M., Lumenta D.B., Mittlböck M., Kamolz L.P. (2013). The use of a polylactide-based copolymer as a temporary skin substitute in deep dermal burns: 1-year follow-up results of a prospective clinical noninferiority trial. Wound Repair. Regen..

[33] Chung K.C., Pillsbury M.S., Walters M.R., Hayward R.A. (1998). Reliability and validity testing of the Michigan Hand Outcomes Questionnaire. J. Hand Surg. Am..

[34] Draaijers L.J., Tempelman F.R.H., Botman Y.A.M., Tuinebreijer W.E., Middelkoop E., Kreis R.W., Van Zuijlen P.P. (2004). The Patient and Observer Scar Assessment Scale: A Reliable and Feasible Tool for Scar Evaluation. Plast. Reconstr. Surg..

[35] Ryan C.M., Parry I., Richard R. (2017). Functional Outcomes Following Burn Injury. J. Burn Care Res..

[36] Jeschke M.G., van Baar M.E., Choudhry M.A., Chung K.K., Gibran N.S., Logsetty S. (2020). Burn injury. Nat. Rev. Dis. Primers.

[37] Holavanahalli R.K., Helm P.A., Gorman A.R., Kowalske K.J. (2007). Outcomes After Deep Full-Thickness Hand Burns. Arch. Phys. Med. Rehabil..

[38] Buta M.R., Donelan M.B. (2024). Evolution of Burn Care. Clin. Plast. Surg..

[39] Kenny E.M., Lagziel T., Hultman C.S., Egro F.M. (2024). Skin Substitutes and Autograft Techniques. Clin. Plast. Surg..

[40] Dargan D., Himmi G., Anwar U., Jivan S., Muthayya P. (2023). A comparison of the epidemiology of isolated and non-isolated hand burns. Burns.

[41] Xie B., Xiao S.C., Zhu S.H., Xia Z.F. (2012). Evaluation of long term health-related quality of life in extensive burns: A 12-year experience in a burn center. Burns.

[42] Bilwani P.K. (2013). Unfavourable results in acute burn management. Indian. J. Plast. Surg..

[43] Aghajanzade M., Momeni M., Niazi M., Ghorbani H., Saberi M., Kheirkhah R., Rahbar H., Karimi H. (2019). Effectiveness of incorporating occupational therapy in rehabilitation of hand burn patients. Ann. Burn. Fire Disasters.

[44] Renneberg B., Ripper S., Schulze J., Seehausen A., Weiler M., Wind G., Hartmann B., Germann G., Liedl A. (2014). Quality of life and predictors of long-term outcome after severe burn injury. J. Behav. Med..

[45] Mata-Ribeiro L., Vieira L., Vilela M. (2022). Epidemiology and Outcome Assessment of Hand Burns: A 3-Year Retrospective Analysis in A Burn Unit. Ann. Burn. Fire Disasters.

[46] Chapman T.T., Richard R.L., Hedman T.L., Chisholm G.B., Quick C.D., Baer D.G., Dewey W.S., Jones J.S., Renz E.M., Barillo D.J. (2008). Military Return to Duty and Civilian Return to Work Factors Following Burns with Focus on the Hand and Literature Review. J. Burn Care Res..

[47] van Baar M.E. (2020). Epidemiology of Scars and Their Consequences: Burn Scars. Textbook on Scar Management.

[48] Quadlbauer S., Pezzei C., Jurkowitsch J., Kolmayr B., Simon D., Rosenauer R., Salminger S., Keuchel T., Tichy A., Hausner T. (2022). Immediate mobilization of distal radius fractures stabilized by volar locking plate results in a better short-term outcome than a five week immobilization: A prospective randomized trial. Clin. Rehabil..

[49] Dyster-Aas J., Kildal M., Willebrand M. (2007). Return to work and health-related quality of life after burn injury. J. Rehabil. Med..

[50] Byrne M., O’Donnell M., Fitzgerald L., Shelley O.P. (2016). Early experience with fat grafting as an adjunct for secondary burn reconstruction in the hand: Technique, hand function assessment and aesthetic outcomes. Burns.

[51] (1996). Synaesthesia in phantom limbs induced with mirrors. Proc. R. Soc. Lond. B Biol. Sci..

[52] Mengistu N.D., Obsa M.S., Gemeda L.A. (2018). Burn Pain Management at Burn Unit of Yekatit 12 Hospitals, Addis Ababa. Pain. Res. Treat..

[53] Howland N., Lopez M., Zhang A.Y. (2016). Pain and Hand Function. Hand Clin..

