# Supplementary material for: Long-Term Functional Outcomes after Hand Burns: A Monocentric Controlled Cohort Study
Source: J Clin Med. 2024 Jun 15;13(12):3509. doi: 10.3390/jcm13123509 (PMC11204761; doi:10.3390/jcm13123509)
Supplement: Supplementary file 1 [file jcm-13-03509-s001.zip › jcm-3047360-supplementary.pdf]

Supplementary

Long-Term Functional Outcomes after Hand Burns:

A Monocentric Controlled Cohort Study

Supplementary S1: Long-term Quick-DASH and MHQ scores comparison of healthy matched control group between patients with hand burns.

	Control Group	Hand Burn Group	p-value
**Quick-DASH, mean (SD)**			
Total score	13.2 (±3.4)	18.0 (±8.5)	**<0.001**
Work	4.6 (±1.5)	6.0 (±3.1)	**0.021**
Sport	5.4 (±2.9)	6.4 (±3.2)	0.148
**MHQ, mean (SD)**			
Function R	94.8 (±11.1)	85.0 (±15.3)	**0.011**
Function L	93.6 (±12.1)	79.2 (±22.7)	**0.045**
Function B	94.2 (±10.2)	77.0 (±21.4)	**<0.001**
Activities of daily living	98.6 (±4.2)	90.4 (±18.0)	**<0.001**
Activities of daily living B	98.2 (±4.7)	88.3 (±18.8)	**0.012**
Work	96.3 (±10.6)	83.0 (±24.4)	**<0.001**
Pain	6.0 (±10.9)	19.4 (±22)	**<0.001**
Aesthetic R	96.2 (±11.6)	86.3 (±23.0)	0.069
Aesthetic L	96.1 (±11.2)	76.0 (29.2)	**0.029**
Aesthetic B	96.2 (±10.5)	71.6 (30.7)	**<0.001**
Satisfaction R	95.1 (±11.6)	81.8 (21.4)	**0.012**
Satisfaction L	94.3 (±11.9)	83.7 (25.6)	**0.024**
Satisfaction B.	94.7 (±2.2)	73.0 (±27.5)	**<0.001**
Overall R/L	95.8 (±7.2)	85.3 (±15.7)	**<0.001**
Overall B	95.6 (±7.2)	76.1 (±19.3)	**<0.001**

SD: standard deviation; R: right hand affected; L: Left hand affected; B: both hands affected. Two-tailed t-test was used.

Supplementary S2: Long-term Quick-DASH and MHQ scores comparison of healthy matched control group between patients with superficial and deep hand burns.

	Control Group	Superficial Hand Burn	Deep Hand Burn	p-ANOVA	p-value Control vs. Superficial	p-value Control vs. Deep	p-value Superficial vs. Deep
**Quick-DASH, mean (SD)**							
Total score	13.2 (±3.4)	15.09 (±7.5)	18.8 (±8.7)	**<0.001**	1.000	**<0.001**	0.252
Work	4.6 (±1.5)	4.3 (±0.7)	6.4 (±3.4)	**0.002**	1.000	**0.003**	0.060
Sport	5.4 (±2.9)	5.2 (±2.5)	6.8 (±3.3)	0.144	1.000	0.195	0.543
**MHQ, mean (SD)**							
Function R	94.8 (±11.1)	86.5 (±18.8)	77.3 (±22.5)	**<0.001**	0.342	**<0.001**	0.264
Function L	93.6 (±12.1)	88.1 (±17.7)	81.8 (±25.1)	**0.006**	1.000	**0.005**	0.868
Function B	94.2 (±10.2)	78.2 (±24.9)	76.4 (±20.5)	**<0.001**	**0.013**	**<0.001**	1.000
Activities of daily living	98.6 (±4.2)	92.2 (±18.0)	89.7 (±18.3)	**0.002**	0.330	**0.002**	0.657
Activities of daily living B	98.2 (±4.7)	86.1 (±24.5)	89.2 (±16.6)	**<0.001**	**0.013**	**0.007**	1.000
Work	96.3 (±10.6)	82.7 (±26.9)	82.7 (±24.1)	**<0.001**	0.059	**<0.001**	1.000
Pain	6.0 (±10.9)	16.2 (±23.8)	20.7 (±21.7)	**<0.001**	0.169	**<0.001**	1.000
Aesthetic R	96.2 (±11.6)	82.7 (±31.3)	75.9 (±28.2)	**<0.001**	0.129	**<0.001**	0.978
Aesthetic L	96.1 (±11.2)	82.2 (±30.6)	80.1 (±26.7)	**<0.001**	0.087	**<0.001**	1.000
Aesthetic B	96.2 (±10.5)	73.0 (±36.6)	70.9 (±29.0)	**<0.001**	**0.005**	**<0.001**	1.000
Satisfaction R	95.1 (±11.6)	85.6 (±22.7)	76.3 (±24.5)	**<0.001**	0.293	**<0.001**	0.346
Satisfaction L	94.3 (±11.9)	84.6 (±23.5)	80.1 (±29.3)	**0.003**	0.419	**0.003**	1.000
Satisfaction B	94.7 (±2.2)	77.6 (±27.0)	71.1 (±28.1)	**<0.001**	**0.030**	**<0.001**	1.000
Overall R/L	95.8 (±7.2)	97.8 (±2.2)	82.7 (±16.1)	**<0.001**	1.000	**<0.001**	**0.011**
Overall B	95.6 (±7.2)	75.7 (21.5)	76.2 (±18.9)	**<0.001**	**<0.001**	**<0.001**	1.000

SD: standard deviation; R: right hand affected; L: Left hand affected; B: both hands affected. One-way ANOVA with Bonferroni-adjustment was used.

Supplementary S3: Long-term Quick-DASH and MHQ scores comparison of healthy matched control group between patients with bilaterally and unilaterally affected hand burns.

	Control Group	Unilateral Hand Burn	Bilateral Hand Burn	p-ANOVA	p-value Control vs. Unilateral	p-value Control vs. Bilateral	p-value Unilateral vs. Bilateral
**Quick-DASH, mean (SD)**							
Total score	13.2 (±3.4)	17.4 (±8.5)	18.5 (±8.6)	**<0.001**	**0.009**	**0.002**	1.000
Work	4.6 (±1.5)	6.1 (±3.7)	5.7 (±2.5)	**0.037**	0.051	0.323	1.000
Sport	5.4 (±2.9)	5.6 (±2.3)	7.2 (±3.7)	0.107	1.000	0.116	0.367
**MHQ**							
Function R	94.8 (±11.1)	83.5 (±20.4)	73.9 (±22.6)	**<0.001**	**0.006**	**<0.001**	0.089
Function L	93.6 (±12.1)	86.5 (±21.9)	78.9 (±25.1)	**0.003**	0.234	**0.002**	0.342
Function B	94.2 (±10.2)	NA	76.9 (±21.4)	NA	NA	**<0.001**	NA
Activities of daily living	98.6 (±4.2)	91.8 (±18.1)	NA	NA	**<0.001***	NA	NA
Activities of daily living B	98.2 (±4.7)	NA	88.3 (±18.8)	NA	NA	**0.012**	NA
Work	96.3 (±10.6)	86.9 (±20.8)	77.9 (±27.9)	**<0.001**	0.058	**<0.001**	0.190
Pain	6.0 (±10.9)	14.3 (±16.6)	25.9 (±26.2)	**<0.001**	0.070	**<0.001**	**0.025**
Aesthetic R	96.2 (±11.6)	84.6 (±24.1)	69.1 (±31.8)	**<0.001**	**0.031**	**<0.001**	**0.015**
Aesthetic L	96.1 (±11.2)	86.7 (±21.9)	74.1 (±31.4)	**<0.001**	0.101	**<0.001**	0.054
Aesthetic B	96.2 (±10.5)	NA	71.6 (±30.7)	NA	NA	**<0.001**	NA
Satisfaction R	95.1 (±11.6)	84.7 (±19.6)	70.9 (±27.5)	**<0.001**	**0.026**	**<0.001**	**0.013**
Satisfaction L	94.3 (±11.9)	86.5 (±24.1)	75.0 (±30.8)	**<0.001**	0.263	**<0.001**	0.112
Satisfaction B	94.7 (±2.2)	NA	72.9 (±27.5)	NA	NA	**<0.001**	NA
Overall R/L	95.8 (±7.2)	85.3 (±15.7)	NA	NA	**<0.001***	NA	NA
Overall B	95.6 (±7.2)	NA	76.1 (±19.3)	NA	NA	**<0.001**	NA

SD: standard deviation; R: right hand affected; L: Left hand affected; B: both hands affected; NA: not applicable. One-way ANOVA with Bonferroni-adjustment and two-tailed t-test were used.

Supplementary S4: Long-term Quick-DASH and MHQ scores comparison of healthy matched control group between patients with isolated and non-isolated hand burns.

	Control Group	Isolated Hand Burn	Non-isolated Hand Burn	p-ANOVA	p-value Control vs. isolated	p-value Control vs. non-isolated	p-value isolated vs. non-isolated
**Quick-DASH, mean (SD)**							
Total score	13.2 (±3.4)	21.6 (±12.2)	17.1 (±7.4)	**<0.001**	**<0.001**	**0.004**	0.131
Work	4.6 (±1.5)	8 (±5.7)	5.5 (±2.2)	**0.002**	**<0.001**	0.316	**0.030**
Sport	5.4 (±2.9)	7 (±3.3)	6.3 (±3.2)	0.317	0.819	0.628	1.000
**MHQ, mean (SD)**							
Function R	94.8 (±11.1)	82.0 (±14.4)	78.7 (±22.9)	**<0.001**	0.092	**<0.001**	1.000
Function L	93.6 (±12.1)	85.0 (±26.6)	82.7 (±23.2)	**0.009**	0.599	**0.008**	1.000
Function B	94.2 (±10.2)	NA	76.9 (±21.4)	NA	NA	**<0.001**	NA
Activities of daily living	98.6 (±4.2)	87.1 (±26.5)	91.1 (±16.1)	**0.002**	**0.030**	**0.007**	1.000
Activities of daily living B	98.2 (±4.7)	NA	88.3 (±18.8)	NA	NA	**0.012**	NA
Work	96.3 (±10.6)	78.0 (±28.5)	83.9 (±23.7)	**<0.001**	**0.015**	**0.002**	1.000
Pain	6.0 (±10.9)	15.5 (±19.2)	20.2 (±22.6)	**<0.001**	0.330	**<0.001**	1.000
Aesthetic R	96.2 (±11.6)	89.3 (±10.6)	75.4 (±30.5)	**<0.001**	1.000	**<0.001**	0.187
Aesthetic L	96.1 (±11.2)	87.5 (±14.6)	79.9 (±28.7)	**<0.001**	0.799	**<0.001**	1.000
Aesthetic B	96.2 (±10.5)	NA	71.6 (±30.7)	NA	NA	**<0.001**	NA
Satisfaction R	95.1 (±11.6)	73.4 (±22.4)	79.7 (±24.6)	**<0.001**	**0.003**	**<0.001**	1.000
Satisfaction L	94.3 (±11.9)	83.3 (±32.9)	80.9 (±27.2)	**0.004**	0.519	**0.003**	1.000
Satisfaction B	94.7 (±2.2)	NA	72.9 (±27.5)	NA	NA	**<0.001**	NA
Overall R/L	95.8 (±7.2)	80.4 (±20.8)	87.3 (±12.9)	**<0.001**	**<0.001**	**0.004**	0.278
Overall B	95.6 (±7.2)	NA	76.1 (±19.3)	NA	NA	**<0.001**	NA

SD: standard deviation; R: right hand affected; L: Left hand affected; B: both hands affected; NA: not applicable. One-way ANOVA with Bonferroni-adjustment and two-tailed t-test were used.

Supplementary S5: Long-term POSAS scores (patient perspective) comparison regarding burn depth, affected hands and (non-)isolated hand burns in patients after hand burns.

	**Superficial Hand Burn**	**Deep Hand Burn**	**p-value**	**Unilateral Hand Burn**	**Bilateral Hand Burn**	**p-value**	**Isolated Hand Burn**	**Non- isolated Hand Burn**	**p-value**
**POSAS (patient), mean (SD)**									
Pain	2.1 (±2.3)	2.2 (±2.2)	0.910	1.9 (±2.1)	2.5 (±2.2)	0.377	3.00 (±3.3)	2.0 (±1.9)	0.420
Itching	2.0 (±2.2)	2.9 (±2.5)	0.327	2.5 (±2.3)	3.3 (±2.6)	0.233	4.1 (±3.0)	2.6 (±2.3)	0.077
Colour	3.6 (±2.7)	4.9 (±3.1)	0.290	4.6 (±3.32)	4.8 (±2.9)	0.782	4.7 (+3.7)	4.7 (±3.0)	0.995
Stiffness	3.1 (±3.8)	4.5 (±2.9)	0.267	4.0 (±3.1)	4.6 (±3.1)	0.464	5.2 (±3.2)	4.1 (±3.0)	0.317
Thickness	2.6 (±2.8)	4.8 (±3.1)	0.086	4.2 (±3.3)	4.7 (±3.0)	0.545	4.7 (±3.3)	4.4 (±3.2)	0.796
Irregularity	3.0 (±3.6)	5.2 (±3.0)	0.088	4.6 (±3.2)	5.1 (±3.1)	0.573	5.1 (±3.4)	4.7 (±3.1)	0.758
Overall opinion	3.7 (±3.1)	4.7 (±2.7)	0.383	4.3 (±2.7)	4.8 (±2.7)	0.516	4.3 (±3.3)	4.6 (±2.6)	0.779
Total scores	20.1 (±18.4)	29.3 (±15.9)	0.250	26.1 (±16.6)	29.8 (±16.4)	0.417	31.1 (±21.1)	27.1 (±15.5)	0.507

POSAS: Patient and Observer Scar Assessment Scale; SD: standard deviation. Two-tailed t-test was used.
